# Causal Network Inference for Neural Ensemble Activity

**DOI:** 10.1007/s12021-020-09505-4

**Published:** 2021-01-04

**Authors:** Rong Chen

**Affiliations:** Department of Diagnostic Radiology and Nuclear Medicine, University of Maryland School of Medicine, 22 South Greene Street, Baltimore, MD 21201 USA

**Keywords:** Causal discovery, Neuroimaging, Dynamic Bayesian network, Clustering

## Abstract

Interactions among cellular components forming a mesoscopic scale brain network (microcircuit) display characteristic neural dynamics. Analysis of microcircuits provides a system-level understanding of the neurobiology of health and disease. Causal discovery aims to detect causal relationships among variables based on observational data. A key barrier in causal discovery is the high dimensionality of the variable space. A method called Causal Inference for Microcircuits (CAIM) is proposed to reconstruct causal networks from calcium imaging or electrophysiology time series. CAIM combines neural recording, Bayesian network modeling, and neuron clustering. Validation experiments based on simulated data and a real-world reaching task dataset demonstrated that CAIM accurately revealed causal relationships among neural clusters.

## Introduction

Increasing experimental and computational evidence supports the existence of a specific pattern of connectivity among adjacent neurons during cognition and emotion (Yoshimura and Callaway 2005; Yoshimura et al. 2005; Song et al. 2005; Ko et al. 2013; Litwin-Kumar and Doiron 2012). Interactions among cellular components forming a mesoscopic scale brain network (microcircuit) display characteristic neural dynamics. A microcircuit lies at the heart of the information processing capability of the brain. It carries out a specific computation of a region. Microcircuits have been shown to encode sensory input (Luczak et al. 2007), motor function (Churchland et al. 2007), spatial maps in the entorhinal cortex (Hafting et al. 2005), and behavior choice (Harvey et al. 2012). Analysis of microcircuits provides a system-level understanding of the neurobiology of health and disease.

Calcium imaging (Kerr and Nimmerjahn 2012; Ghosh et al. 2011; Scott et al. 2013) and electrophysiology with electrodes are powerful ways to study microcircuits, leading to an understanding of network architecture of behavior, cognition, and emotion (Ko et al. 2013; Barbera et al. 2016). In contrast to the experimental advances in neural recording techniques, computational analysis of ensemble neural activities is still emerging. A fundamental problem in microcircuit analysis is causal discovery. Causal discovery aims to reveal causal structures by analyzing observational data. Several computational methods have been developed to infer causal networks from ensemble neural activity, including Granger causality (Chen et al. 2006; Hu et al. 2018) and conditional independence inference based on dynamic Bayesian networks (DBNs) (Eldawlatly et al. 2010).

A key barrier in causal discovery from multiple time series is high dimensionality. For example, calcium imaging can observe ensemble neural activity of hundreds of neurons. Naively applying causal discovery algorithms to such high-dimensional data causes several problems. First, this naïve approach ignores the intrinsic hierarchical structure of the microcircuit. Neurons often form clusters and neurons in the same cluster have similar functional profiles. For example, D1- and D2-medium spiny neurons (MSNs) in the dorsal striatum are grouped into spatially compact clusters (Barbera et al. 2016). In the visual cortex, highly connected neurons in a cortical column receive similar visual input (Yoshimura et al. 2005). These studies suggest that neurons in a microcircuit form clusters (or modules, communities). Second, constructing a model from such high-dimensional data with a cluster structure often leads to overfitting (Hastie et al. 2009), an unstable model (Sauerbrei et al. 2011; Chen and Herskovits 2007), and poor parameter estimation (Chen and Herskovits 2007).

The proposed method, called Causal Inference for Microcircuits (CAIM), aims to reconstruct causal mesoscopic-scale networks from observational calcium imaging or electrophysiology time series. CAIM combines neural recording, Bayesian network modeling, and neuron clustering. To address the high-dimensionality problem, CAIM utilizes clustering to group neurons into clusters. To solve the causal discovery problem, CAIM uses DBNs to identify conditional independence. CAIM enables us to move toward a circuit-based approach to understand the brain, in which a behavior is understood to result from specific spatiotemporal patterns of circuit activity related to specific neuronal populations.

This paper is organized as follows. "Background and Related Work" describes the background and related work. "Method" provides the CAIM algorithm, including neuron clustering and causal network inference. In "Results", validation experiments on simulated neural activity data and application of CAIM to a real-world dataset are presented. "Discussion" includes the discussion and issues requiring further investigation are provided, which are followed by conclusions.

## Background and Related Work

Network analysis (or connectivity analysis) methods for neural signals can be classified as synchrony analysis and causal discovery. In synchrony analysis, an undirected graph is generated. Synchrony has been extensively studied in neuroscience (Averbeck et al. 2006). Correlation, partial correlation, and mutual information have been used to measure the association between a pair of neurons.

The gold standard of establishing a causal relationship is performing planned or randomized experiments (Fisher 1970). Pearl proposed an intervention-based framework for causality analysis (Pearl 2009) and distinguished the observational conditional probability *P*(*Y*|*X*) and interventional conditional probability P(*Y*|do(*X*)) where the do(.) operator is an intervention. The notion of intervention by Pearl implies that if we manipulate *X* and nothing happens, then *X* is not the cause of *Y*; otherwise, *X* is one of the causes of *Y*. However, in many scenarios, experiments are too expensive, or not feasible or ethical to carry out. *Causal discovery* (or effective connectivity analysis) aims to infer cause-effect relations among variables based on *observational data*. Granger proposed a framework to infer causality based on prediction improvement (Granger 1969). An important framework of causal discovery is based on conditional independence (Spirtes et al. 2001). This framework considers the dependence between two variables *X* and *Y* given a set of variables ***Z***. Let *X* ⫫ *Y* | ***Z*** denote that *X* and *Y* are conditionally independent given ***Z.*** *X* is not the cause of *Y* if *X*_*t*_ ⫫ *Y*_*t* + 1_ | ***Z***_*t*_. For a set of variables ***V*** = {*X*_1_, …, *X*_*p*_}, a causal graphical model is *G* = (***V***, ***E***), where an edge *X*_*i*_ → *X*_*j*_ represents *X*_*i*_ is a direct cause of *X*_*j*_ relative to variables in ***V***, and *G* is a directed acyclic graph. The assumptions which often are used to relate causal structures to probability densities are the causal Markov assumption, the causal faithfulness assumption, and the causal sufficiency assumption (Spirtes et al. 2001). Under these assumptions, a remarkable result according to Geiger and Pearl (Geiger and Pearl 1990) and Meek (Meek 1995) is the Markov completeness theorem: for linear Gaussian and for multinomial causal relations, an algorithm that identifies the Markov equivalent class is complete (that is, it extracts all information about the underlying causal structure).

There are many studies of causal discovery from multiple time series from problem domains which are not neuroscience-related, such as inferring gene regulatory networks using time-series gene expression data (Bar-Joseph et al. 2012). A kind of inference framework is growth-shrink. Such methods first calculate pairwise associations between **s**_*t +* 1_ and **s**_*t*_; and then remove redundant or spurious connections (Meyer et al. 2007). An example of a growth-shrink based method is MRNET (Meyer et al. 2007), which uses mutual information between variables and minimum-redundancy-maximum-relevance to infer networks. Another kind of inference framework considers network inference as a regression problem and uses ensemble learning to construct the network. BTNET (Park et al. 2018) is an ensemble learning-based method that uses a boosted tree to construct the predictive model.

## Method

CAIM aims to infer causal relationships based on observational calcium imaging or electrophysiology time series. In CAIM, microcircuits are DBNs (Koller and Friedman 2009) representing causal relationships. In a DBN, nodes are variables of interest, and edges (links) represent interactions among variables. If a set of nodes, *π*_*i*_, causally affects the activity of node *i*, then there exists a link from the nodes in *π*_*i*_ to node *i*. *π*_*i*_ is referred to as the parent set of node *i*. Each node is associated with a binary variable which represents whether the node is activated. Each node is associated with an updating rule that specifies how its state changes over time due to the activation of the parent set. Network dynamics are determined by these updating rules. DBNs can characterize system dynamics, handle noisy data, describe locally interacting processes, and support causal inference (Chen et al. 2012).

CAIM infers causal networks from neural ensemble activities. Neural activities can be recorded by calcium imaging or electrophysiology with electrodes. Preprocessing algorithms generate binary neuronal events (spike trains or calcium transient events). A preprocessing pipeline (Barbera et al. 2016) can be used to preprocess calcium imaging data, including image registration, cell mask detection, and neuronal event detection. However, preprocessing is not the focus of CAIM. Let *P* and *T* denote the number of neurons and the number of time points, respectively. The preprocessing step results in **s**_1:T._ For neuron *i*, *s*_*i,t*_ = 1 indicates a neuronal event of neuron *i* at time point *t*, while *s*_*i,t*_ = 0 indicates no event. **s**_*t*_ = [*s*_1,*t*_*, …, s*_*P,t*_] is a *P*-dimensional vector representing neural events of all neurons at time point *t*. **s**_1:T_ = (**s**_1_, …, **s**_*T*_) represents neural activity for all time points.

**Fig. 1:**
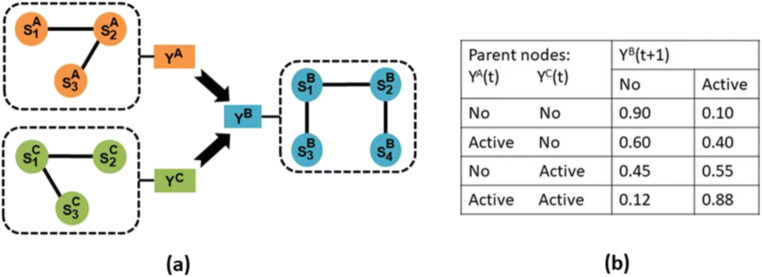
The architecture of CAIM

Figure 1 shows the architecture of CAIM. Figure 1a is the conceptual framework of CAIM. In CAIM, neurons are grouped into clusters. Neurons in the same cluster have similar functional profiles. Each cluster is associated with a latent variable (the cluster state variable) which represents whether the cluster is activated or not. Let *Y*^*A*^(*t*) denote the state variable for cluster *A* at time point *t*. **Y**_*t*_ = [*Y*^*A*^(*t*)*, …, Y*^*Z*^(*t*)] is a vector representing states of all clusters at time point *t*. **Y**_1:T_ = (**Y**_1_, …, **Y**_*T*_) represents cluster states for all time points. Interactions among clusters are described by a DBN. In this DBN, nodes are cluster state variables. The directed temporal interaction between two nodes is represented by a transition probability table (Fig. 1b). For example, Pr(*Y*^*B*^(*t* + 1) = active | *Y*^*A*^(*t*) = active, *Y*^*C*^(*t*) = active) = 0.88 represents activation of cluster *A* and activation of cluster *C* at time point *t* result in the activation of cluster *B* at time point *t* + 1 with probability 0.88.

### Neuron Clustering

The goal of neuron clustering is to group *P* neurons into *K* homogeneous clusters. Coherence, which is pairwise functional association, plays a key role in neural codes (Averbeck et al. 2006; Zohary et al. 1994). Even weak pairwise linear interactions can result in strongly correlated network states in a neural ensemble (Schneidman et al. 2006). Therefore, our clustering algorithm centers on examining coherence. The objects in this clustering problem are neurons. Neurons within each cluster are more similar to each other than neurons assigned to different clusters. Input to neuron clustering is **s**_1:T_. Clustering generates a partition of the variable space. The partition Ω is a vector whose *i*^th^ elements Ω(*i*) is the group membership of neuron *i*.

Neuron clustering is based on the similarity between *s*_*i,*1:T_ and *s*_*j,*1:T_, where *s*_*i,*1:T_ is the observed trajectory of neuron *i*. Therefore, neuron clustering focuses on examining the instantaneous synchrony (the zero-lag synchrony) between neuron pairs. There are many clustering algorithms (Wiwie et al. 2018). *s*_*i,*1:T_ is a trajectory with thousands of observation time points. Each time point is a feature. In this clustering problem, *P* is about several hundred and *T* is several thousand. Therefore, the clustering algorithm needs to handle high-dimensional data. Since we assume that an object belongs to a single cluster, we don’t use fuzzy clustering such as c-Means or probabilistic clustering such as Gaussian mixture models.

CAIM uses graph-based clustering. A graph is constructed by using kd-trees to identify the approximate nearest neighbors for each object (Arya et al. 1998). This graph construction algorithm is computationally efficient. Clusters are detected by the walktrap algorithm (Pons and Latapy 2006) for graph-based community detection. The walktrap algorithm finds densely connected subgraphs based on random walks. The algorithm starts by assigning each node to its own community and calculates the distance for every pair of communities. Communities are merged according to the minimum of their distances and the process is repeated. The number of clusters is estimated by the walktrap algorithm. The walktrap algorithm uses the results of random walks to merge separate communities in a bottom-up manner and creates a dendrogram. Then it uses the modularity score to select where to cut the dendrogram. Therefore, the number of clusters is automictically determined by the algorithm.

After generating the partition, cluster state variables are inferred by voting. For cluster *A*, the percentage of neurons in state 1 at time point *t* is calculated. If this percentage is greater than a threshold, then *Y*^*A*^(*t*) = 1; otherwise, *Y*^*A*^(*t*) = 0. Higher threshold results in sparser cluster activation. If the majority voting is adopted, the threshold is 50%.

Given binary cluster state variables, a loading matrix can be calculated to assess the association between cluster state variables and neurons. The loading matrix has *P* rows and *K* columns. The element (*i*, *j*) in this loading matrix is the relative mutual information (Pregowska et al. 2015) between neuron *i* and cluster *j*. The relative mutual information is in [0, 1]. Higher relative mutual information indicates a stronger association between two binary random variables.

### Causal Network Construction

Causal network construction infers a DBN based on **Y**_1:T,_ which is the dataset including cluster states for all time points. A DBN is defined as a pair, (B_1_, B_→_), where B_1_ is a Bayesian network defining the baseline probability distribution; and B_→_ defines the transition probability P(**Y**_*t +* 1_ | **Y**_*t*_). That is, B_→_ is a two-slice temporal Bayesian network (2TBN). The state of node *i* at time point *t +* 1 is determined by the states of its parent set before *t +* 1, and is independent of the states of any other nodes. We use *π*_*i*_ to denote the parent set of node *i*. *π*_*i*_ is a subset of **Y**_*t*_. For example, in Fig. 1b, *Y*^*A*^_*t*_ and *Y*^*C*^_*t*_ determine *Y*^*B*^_*t +* 1_, then *π*_*B*_ *=* (*Y*^*A*^_*t*_, *Y*^*C*^_*t*_).

The DBN-based causal discovery assumes causal sufficiency, the causal Markov condition, and faithfulness (Spirtes et al. 2001). Under these conditions, the causal relationship can be discovered by machine learning algorithms. Our algorithm generates a directed weighted graph *G* modeling the linear/nonlinear interactions among cluster state variables. We use a random forest-based method to find the parent set of a node. For a node *Y*^*A*^_*t +* 1_, we construct a random forest model to predict *Y*^*A*^_*t* + 1_ based on variables in **Y**_*t*_ = [*Y*^*A*^(*t*)*, …, Y*^*Z*^(*t*)]. The implementation is similar to that in (Huynh-Thu et al. 2010). A random forest ensemble is generated to predict *Y*^*A*^_*t* + 1_ based on variables in **Y**_*t*_. In the model ensemble, each tree model is constructed based on a bootstrap sample from the original sample and, at each test node, a subset of variables is selected at random among all candidate variables in **Y**_*t*_ before determining the best split (to divide a node in a tree into two daughter nodes). To quantify the variable importance, for each test node in a tree, we compute the reduction of variance of the output variable due to the split. For a single tree, the importance of a variable is computed by summing the variance reduction values of all tree nodes where this variable is used to split. For a tree ensemble, the importance score of a variable is the average over all trees. Variable importance of *Y*^*B*^_*t*_ is used as the weight for the link *Y*^*B*^_*t*_ → *Y*^*A*^_*t* + 1*.*_ Higher weights represent stronger relationships. Random forests have the capability to model nonlinear and combinational interactions (interactions involving multiple nodes, instead of pairwise) and handle high-dimensional data. In our implementation, we adopt the parameter tuning process of random forest described in (Huynh-Thu et al. 2010).

## Results

We evaluated CAIM on simulated spike trains, data from a biophysics-based simulation, and real-world neural activity data for a delayed reaching task. All experiments were conducted in a workstation with Intel Core i7-4720HQ CPU @2.6GHz (4 cores and 8 virtual cores) and 16G memory.

### Simulated Spike Trains

**Fig. 2:**
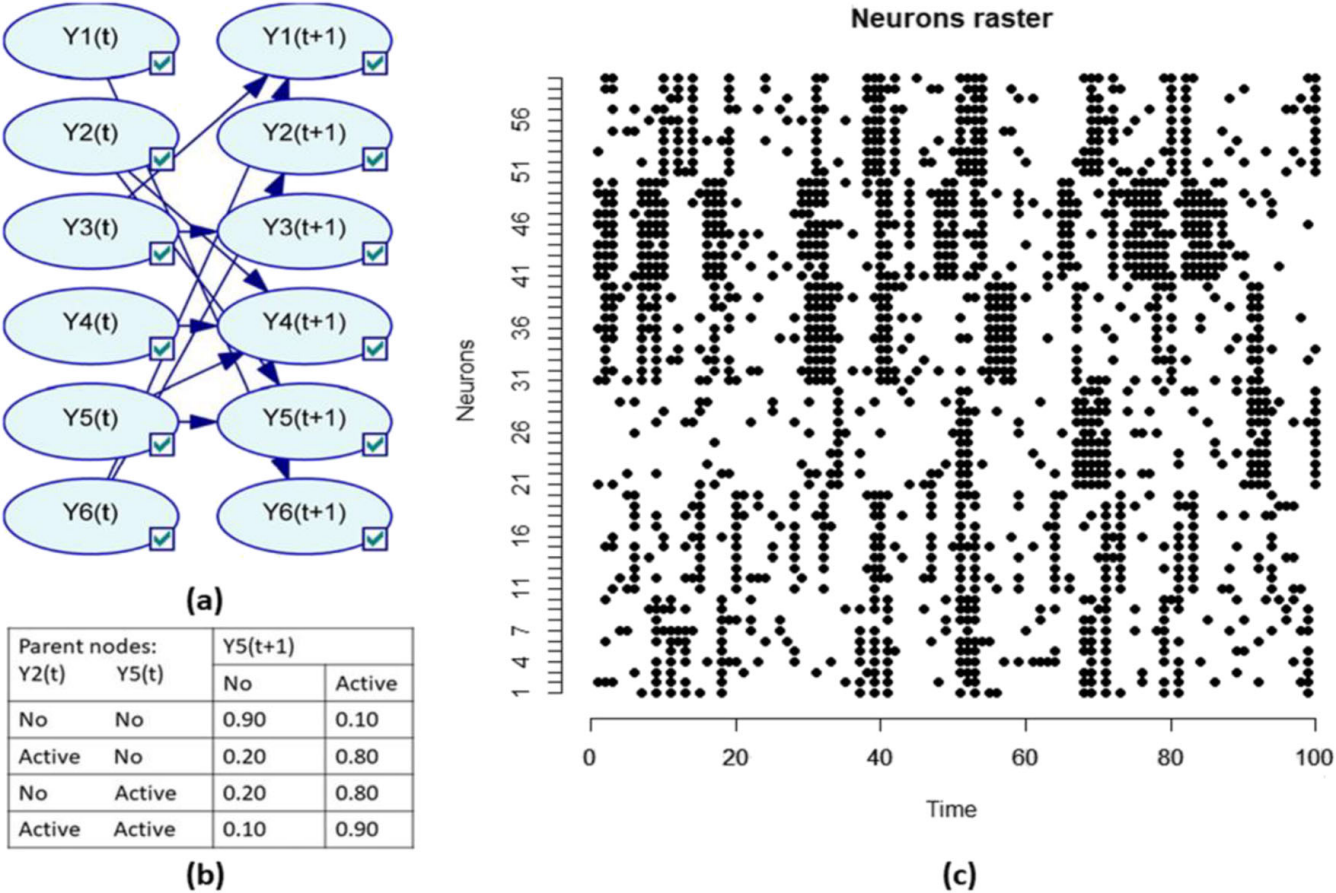
The simulated spike train data. **a** The ground-truth DBN model which describes temporal interactions among cluster state variables. **b** The transition probability table for cluster 5. **c** Spike trains of 60 neurons. Noise level is 0.1

In this experiment, we used simulated binary spike trains to evaluate CAIM. The interactions among clusters were described by a ground-truth DBN G^*^. An example of the structure of G^*^ is depicted in Fig. 2a. Parameters in G^*^ were set to represent additive effects. The transition probability table for node 5 is depicted in Fig. 2b. The data generation process included sampling and neural data generation. In the sampling step, we sampled G^*^ and generated simulated data for cluster states. Let *Y*^*i*^_1:*T*_ be the trajectory of cluster *i*. In neural data generation, the trajectory of a neuron in cluster *i* is generated by flipping the binary state of *Y*^*i*^_1:*T*_ with a probability *λ*. *λ* represented noise level and 1-*λ* characterized the within-cluster homogeneity. We evaluated CAIM for various noise level (subtask 1), cluster similarity (subtask 2), and number of clusters (subtask 3).

To evaluate neuron clustering, we compared CAIM clustering with other clustering methods including K-means, clustering by density peaks, and the Fuzzy c-means (FCM) based method in (Fellous et al. 2004; Toups et al. 2011). K-means defines a cluster as a sphere around the cluster centroid. The number of clusters was estimated by the Calinski-Harabasz index. K-means was randomly initialized 100 times. Clustering by density peaks is based on the idea that cluster centers are characterized by a higher density than the neighbors of centers and by a relatively large distance from objects with higher densities. To detect the cluster structure, we need to manually specify two parameters. In the FCM-based method, we first calculated a *P× P* distance matrix where the (*i*, *j*) element of this matrix is the Manhattan distance between neurons *i* and *j*. Then we applied FCM on the columns of distance matrix. The number of clusters was determined by the gap statistic. Neuron clustering performance was evaluated by two cluster validity indexes: the Silhouette score and Rand index (Ye 2003). Higher Silhouette score or Rand index represents better clustering. The Silhouette score has a range of [−1, 1]. A score near 1 indicates that the sample is far from neighboring clusters, a score of 0 indicates that the sample is on or very close to the decision boundary, and negative values indicate poor assignment. The Rand index determines the similarity between the estimated label and the ground-truth label as a function of positive and negative agreements in pairwise cluster assignments; when two labels agree perfectly, the Rand index is 1.

For causal discovery, we compared our causal network discovery algorithm to Bayesian network structure learning (BNS), Bayesian network structure learning with resampling (BNSR) (Chen et al. 2017), and GLMNET. In BNS, we used the algorithm in (Chen et al. 2012) to detect the parent set of *Y*^*A*^_*t* + 1_. The association among nodes by the Bayesian Dirichelet score (Chen et al. 2012), which is the marginal likelihood or evidence P(*G* | **D**), where **D** is the observed data. The Bayesian Dirichelet score is decomposable. That is, we can maximize this score node by node. For each node *Y*^*i*^_*t* + 1_, we used the algorithm in (Chen et al. 2012) to search for a set of nodes in **Y**_*t*_ which maximizes the Bayesian Dirichelet score. This set of nodes is the parent set of *Y*^*i*^_*t* + 1_. Based on these parent sets, we can generate a graph describing causal interactions. In BNSR, bootstrap resampling was used to stabilize the Bayesian network learning process. We resampled the original dataset 1000 times and utilized BNS to generate a DBN model for each resampled dataset. For an edge *Y*^*B*^_*t*_ → *Y*^*A*^_*t* + 1,_ the edge strength was measured by the frequency of this edge appearing in the model ensemble. In GLMNET, for *Y*^*A*^_*t* + 1_, variables in **Y**_*t*_ which were most predictive of *Y*^*A*^_*t* + 1_ were identified by Lasso and elastic-net regularized generalized linear models (Friedman et al. 2010). Parameters in GLMNET were tuned based on internal cross-validation. To improve model stability, we used bootstrap resampling to resample the raw dataset 1000 times and generated models for resampled datasets. The model ensemble included 1000 models. For a directed link *Y*^*B*^_*t*_ → *Y*^*A*^_*t* + 1,_ the link strength was measured by the frequency of this link appearing in the model ensemble. CAIM, BNSR, GLMNET generated weighted directed graphs. The higher edge weight of *Y*^*B*^_*t*_ → *Y*^*A*^_*t* + 1_ represents a stronger relationship between *Y*^*B*^_*t*_ and *Y*^*A*^_*t* + 1_. BNS generated an unweighted graph.

For causal discovery, we used area under the ROC Curve (AUC) to evaluate algorithms’ performance. AUC was calculated based on the generated graph and the ground-truth DBN. Higher AUC indicated an algorithm achieved better performance in detecting the ground-truth DBN structure.

In subtask 1, we evaluated CAIM with different noise levels. In this subtask, 60 neurons were grouped into 6 clusters. Each cluster had 10 neurons. In the simulation, *T* = 5000 and *P* = 60. The structure of G^*^ is depicted in Fig. 2a. Datasets for three noise levels, 0.1 (low noise level), 0.2 (medium noise level), and 0.3 (high noise level), were generated. The first 100 observations of all neurons for noise level 0.1 are depicted in Fig. 2c. In subtask 2, we evaluated CAIM with different cluster similarity levels. In this subtask, 60 neurons were grouped into 6 clusters (each cluster had 10 neurons). *T* = 5000 and *P* = 60. The structure of G^*^ is depicted in Fig. 2a. Noise level was 0.2. We varied parameters of the ground truth DBNs and generated datasets with different cluster similarity levels. For a dataset, cluster similarity was quantified by the average Hamming distances across all cluster pairs. We generated three datasets: low similarity (Hamming distance = 2696), middle similarity (Hamming distance = 1461), and high similarity (Hamming distance = 862). Higher similarity is more challenging for neuron clustering. In subtask 3, we evaluated CAIM with different cluster numbers. In this subtask, each cluster had 10 neurons. We generated datasets with 3 clusters (30 neurons), 6 clusters (60 neurons), and 9 clusters (90 neurons). The structure of G^*^ was randomly generated. Noise level was 0.2.

**Fig. 3:**
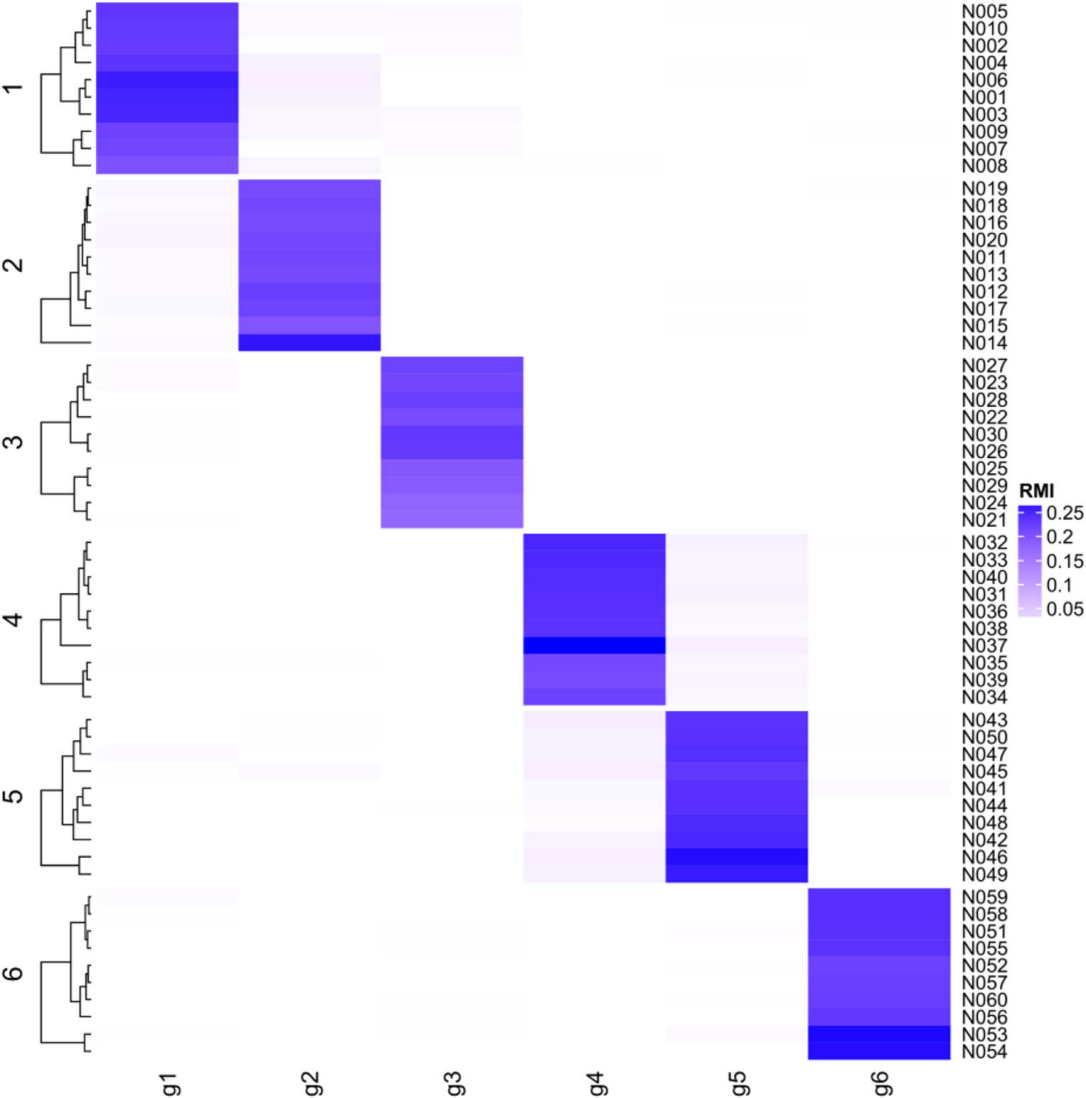
The loading matrix of neuron clustering for subtask 1. Noise level is 0.3

Neuron clustering results for subtask 1 are summarized in Table 1. Figure 3 depicts the loading matrix of neuron clustering for noise level 0.3. For all noise levels, CAIM achieved the best clustering performance. CAIM always detected the correct number of clusters and identified the correct cluster structure (Rand index = 1). Neuron clustering results for subtask 2 are summarized in Table 2. For different cluster similarity levels, CAIM consistently detected the corrected number of clusters and identified the correct cluster structure. Neuron clustering results for subtask 3 are summarized in Table 3. For varying cluster numbers, CAIM detected the corrected number of clusters and identified the correct cluster structure. For all experimental conditions, CAIM and FCM consistently achieved higher Silhouette score and Rand index than did K-means and clustering by density peaks. Overall, CAIM achieved the highest Silhouette score and Rand index.

**Table 1:** Clustering results for the simulated spike trains with different noise levels

Noise level	Method	Detected number of clusters	Silhouette score	Rand index
0.1	CAIM	6	0.268	1.000
	K-means	6	0.268	1.000
	Clustering by density peaks	7	0.075	0.594
	FCM	6	0.268	1.000
0.2	CAIM	6	0.113	1.000
	K-means	2	0.062	0.259
	Clustering by density peaks	4	0.039	0.488
	FCM	6	0.113	1.000
0.3	CAIM	6	0.043	1.000
	K-means	2	0.024	0.259
	Clustering by density peaks	6	0.013	0.502
	FCM	10	0.015	0.748

**Table 2:** Clustering results for the simulated spike trains with different cluster similarities

Cluster similarity	Method	Detected number of clusters	Silhouette score	Rand index
Low	CAIM	6	0.145	1.000
	K-means	2	0.117	0.259
	Clustering by density peaks	5	0.119	0.631
	FCM	6	0.145	1.000
Middle	CAIM	6	0.108	1.000
	K-means	2	0.065	0.259
	Clustering by density peaks	4	0.038	0.488
	FCM	6	0.108	1.000
High	CAIM	6	0.075	1.000
	K-means	4	0.058	0.550
	Clustering by density peaks	4	0.020	0.414
	FCM	10	0.027	0.748

**Table 3:** Clustering results for the simulated spike trains with varying numbers of clusters

The number of clusters	Method	Detected number of clusters	Silhouette score	Rand index
3	CAIM	3	0.124	1.000
	K-means	3	0.124	1.000
	Clustering by density peaks	5	0.008	0.293
	FCM	3	0.124	1.000
6	CAIM	6	0.109	1.000
	K-means	2	0.062	0.259
	Clustering by density peaks	4	0.043	0.363
	FCM	6	0.109	1.000
9	CAIM	9	0.147	1.000
	K-means	8	0.138	0.876
	Clustering by density peaks	9	0.106	0.823
	FCM	10	0.131	0.966

**Fig. 4:**
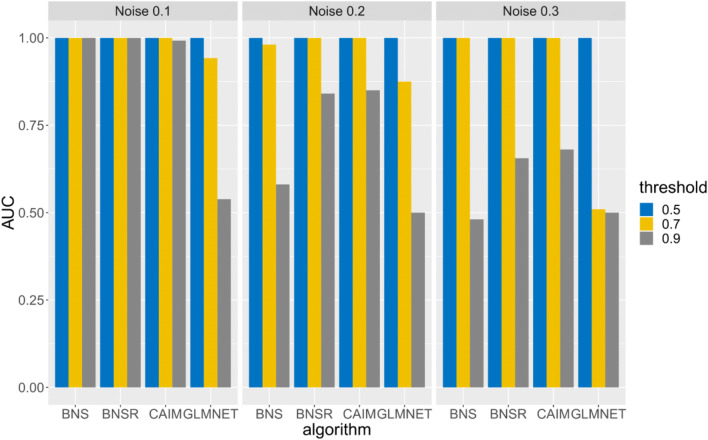
AUCs of BNS, BNSR, CAIM, and GLMNET for the simulated spike train data with varying noise levels

**Fig. 5:**
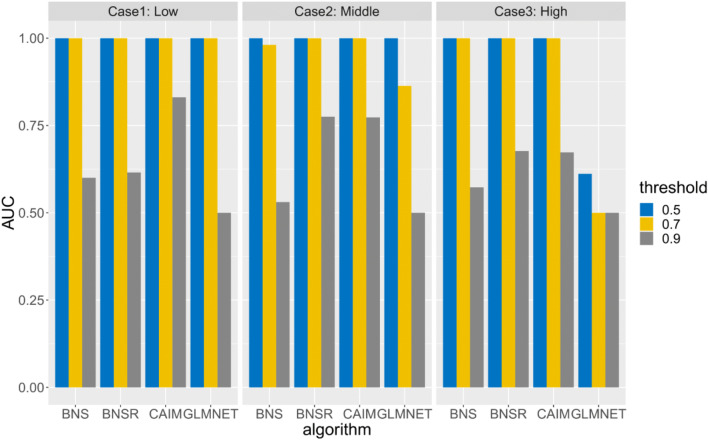
AUCs of BNS, BNSR, CAIM, and GLMNET for the simulated spike train data with varying cluster similarity levels

**Fig. 6:**
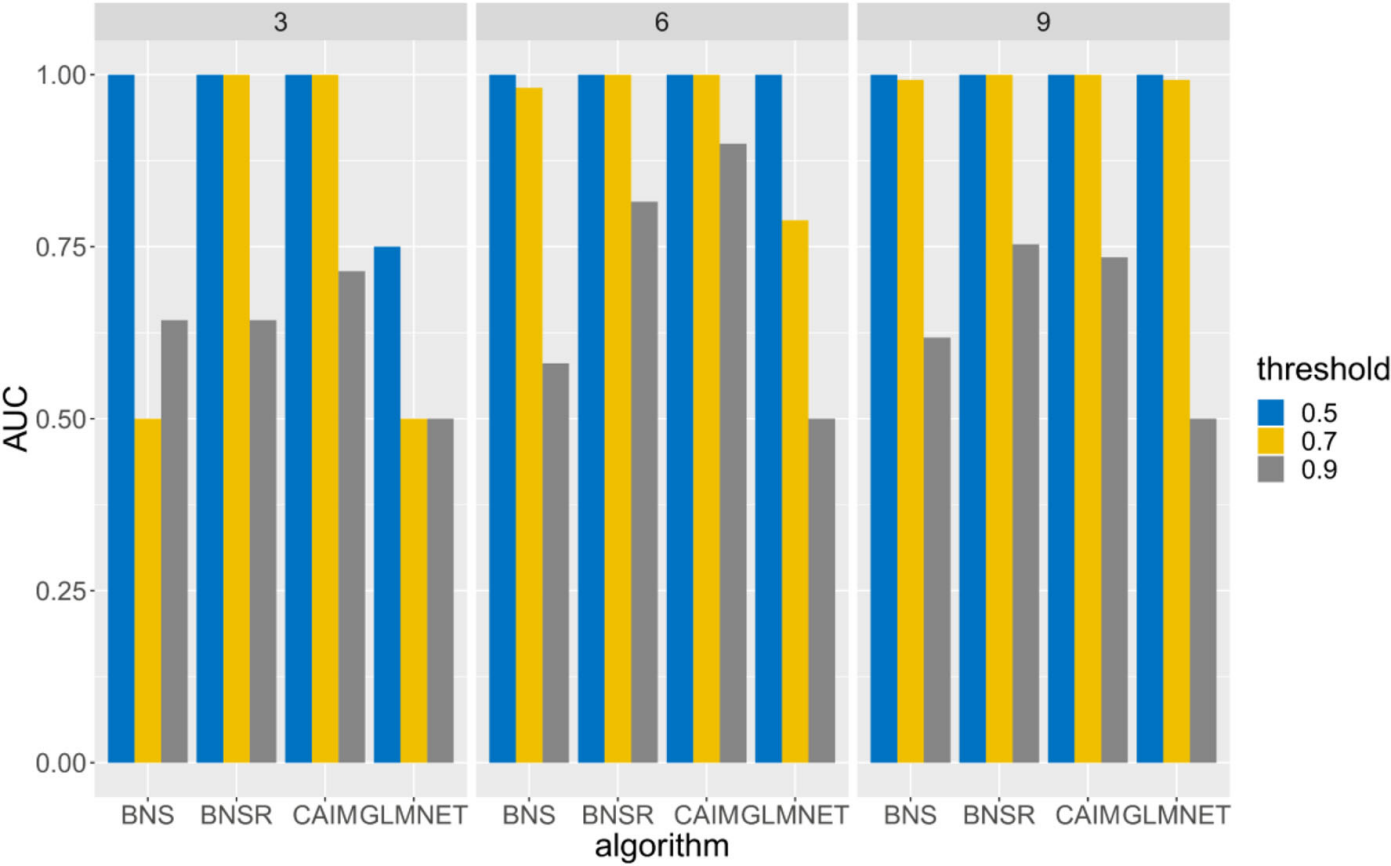
AUCs of BNS, BNSR, CAIM, and GLMNET for the simulated spike train data with varying cluster numbers

Figures 4, 5 and 6 depict the AUCs of BNS, BNSR, CAIM and GLMNET for subtasks 1, 2, and 3, respectively. CAIM achieved the highest AUC in most combinations of experimental setups and thresholds. For threshold = 0.5, CAIM’s AUCs were 1 for all scenarios. CAIM was robust to the threshold to infer binary cluster states. CAIM and BNSR consistently achieved higher AUCs than did BNS and GLMNET. The typical execution time of BNS, BNSR, CAIM and GLMNET were 0.23 s, 13.73 s, 8.28 s, and 1571.92 s. CAIM and BNSR had similar execution time while GLMNET had a much longer execution time. Both AUCs and execution times of BNSR and CAIM were similar, although the AUC of CAIM was consistently higher. Relative to BNS, BNSR achieved significantly higher AUC. This is because BNSR is an ensemble learning based method and achieves consistent estimates by combining solutions from different bootstrap resampled training data sets.

Collectively, these experiments demonstrate that CAIM can detect the cluster structure and achieve the optimal performance balance (high AUC and short running time). We found that CAIM accurately inferred the causal relationships.

### Biophysics Based Simulation

In this experiment, a biophysics-based simulation was used to assess CAIM. The simulation modeled interactions among a set of integrate-and-fire (I&F) neurons with noise. Such a neuron model can represent virtually all postsynaptic potentials or currents described in the literature (e.g. α-functions, bi-exponential functions) (Brette et al. 2007). The neuron model (Gütig and Sompolinsky 2006) is as follows:

1 $$ \frac{dV}{dt}=\frac{\left({V}_{rest}-V\right)}{\tau }+\sigma \times \tau \times \left(-0.5\right)\times \upvarepsilon $$

where *V* is the membrane potential, *V*_*rest*_ is the rest potential, *ε* is a Gaussian random variable with mean 0 and standard deviation 1, *τ* is the membrane time constant, and *σ* is a parameter controlling the noise term. Spikes received through the synapses trigger changes in *V*. A neurons fires if *V* is greater than a threshold. This neuron cannot generate a second spike for a brief time after the first one (refractoriness).

**Fig. 7:**
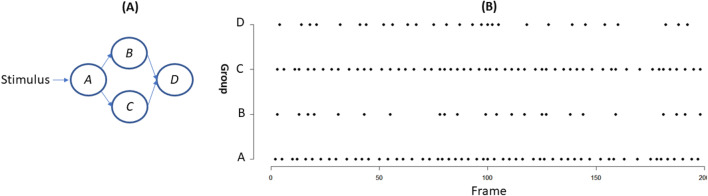
Causal discovery results for the biophysics-based simulation. **a** The ground-truth causal graph. **b** The spike trains of cluster states (the first 200 frames)

Our simulation included 160 neurons in four groups: *A*, *B, C,* and *D*. Each group had 40 neurons. The ground-truth causal graph is depicted in Fig. 7a. Neurons in group *A* had no parent nodes. They all received a stimulus. Neurons in group *B* had two or three neurons in group *A* as parent nodes. Neurons in group *C* had two or three neurons in group *A* as parent nodes. If a parent node fired, the membrane potential of the target node increased by *w*. The *w* of connections between *A* and *B* was different from that of *A* and *C*. Firing of neurons in groups *B* and *C* caused firing of neurons in group *D.* The simulated spike trains are depicted in Fig. 7b.

CAIM accurately detected the cluster structure with the RAND score 0.98. This weighted graph was robust to the threshold to infer binary cluster states and remained stable for the threshold in [0.3 0.7]. We chose threshold = 0.5. The edge weight had a bimodal distribution. The edge weights of *Y*^*A*^_*t*_ → *Y*^*B*^_*t* + 1_, *Y*^*A*^_*t*_ → *Y*^*C*^_*t* + 1_, *Y*^*B*^_*t*_ → *Y*^*D*^_*t* + 1_, and *Y*^*C*^_*t*_ → *Y*^*D*^_*t* + 1_ were 0.90, 0.90, 0.50, and 0.47, respectively. Other edges had very low weights. The strong links characterize the strong causal relationship in Fig. 7a. Overall, CAIM was able to identify the causal relationship between these neuron groups.

### Real-World Neural Activity Data for a Delayed Reaching Task

CAIM was evaluated based on a spike dataset acquired during the delay period in a standard delayed reaching task (Santhanam et al. 2009). A male rhesus monkey performed a standard instructed-delay center-out reaching task. Animal protocols were approved by the Stanford University Institutional Animal Care and Use Committee. The dataset contains spike trains recorded simultaneously by a silicon electrode array (Cyberkinetics, Foxborough, MA) from 61 neurons in the right premotor cortex. The reaching task dataset contained two experimental conditions (conditions 1 and 2). Each condition had 56 trials. The spike train had a length between 1018 ms and 1526 ms. Spike trains are binned using a non-overlapping bin with a width of 20 ms. This bin size was found to work well for population activity recorded in the motor cortex (Cowley et al. 2012). Among 61 neurons, 16 neurons had a low firing rate (<5 spikes/s) and were excluded from the analysis. Excluding these low firing neurons from causal discovery doesn’t exclude the possibility that they contributed to the observed ensemble activity. We excluded them because these low firing neurons had too few active states needed to firmly establish causal relationships (Chen et al. 2008).

**Fig. 8:**
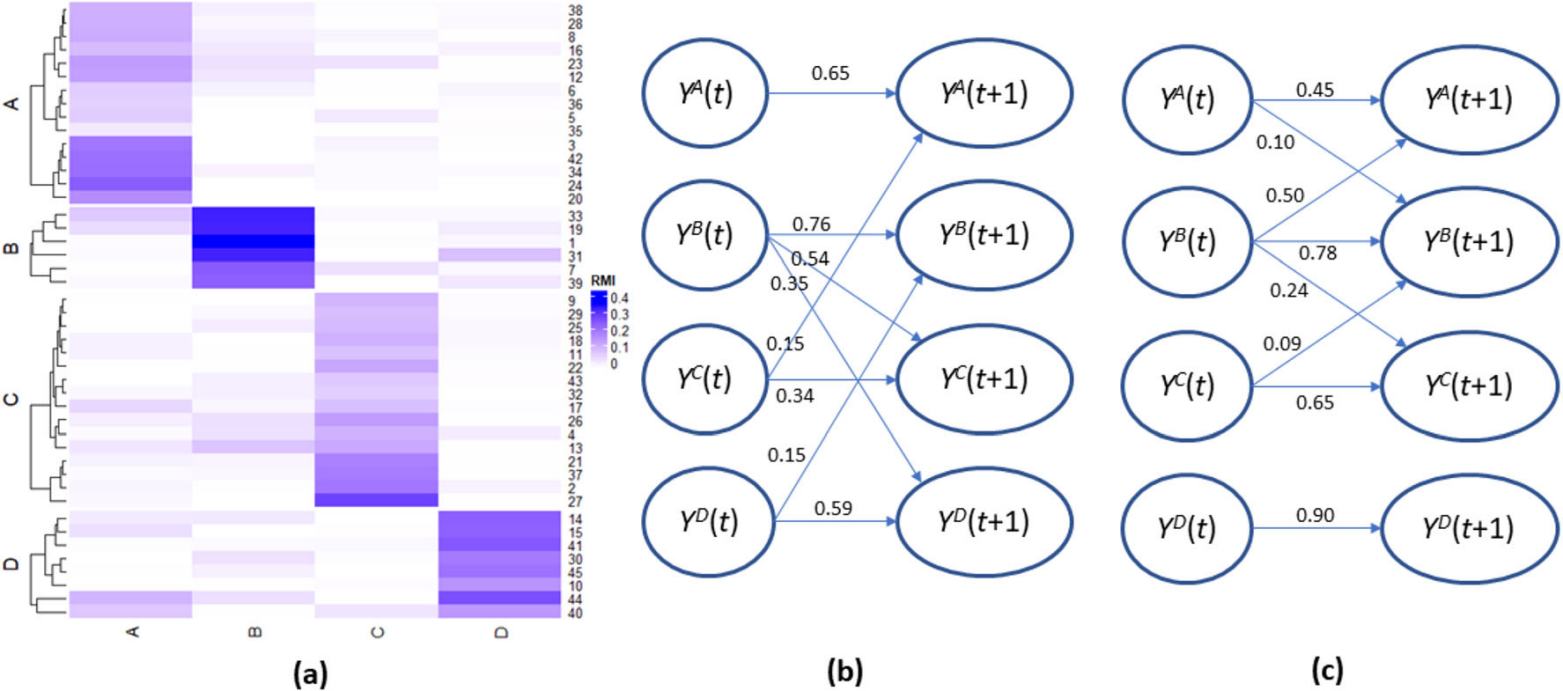
Causal discovery results for the reach-task dataset. **a** The loading matrix for neuron clustering. Rows are neurons (split by the cluster label); and columns are clusters. **b** and **c** are DBNs for condition 1 and 2. In DBNs, edge weights represent strength of connectivity

CAIM found 4 clusters. Figure 8a depicts the loading matrix of neuron clustering. The average within-cluster relative mutual information was 0.187, while the average between-cluster relative mutual information was 0.016. These results demonstrated good cluster separation.

The detected causal networks are depicted in Fig. 8b and c which are the networks for two different conditions, respectively. Strong links (edges with weights greater than the median weight) are shown. Both causal graphs demonstrated persistence. That is, for a cluster, *Y*_*t* + 1_ is driven by *Y*_*t*._ Persistence may reflect continuous firing. The causal graphs for these two conditions also had significant structural differences. In condition 1, *Y*^*A*^_*t* + 1_ was strongly driven by *Y*^*A*^_*t*_ and *Y*^*C*^_*t*_. Such a pattern was changed in condition 2. In condition 2, *Y*^*A*^_*t* + 1_ was driven by *Y*^*A*^_*t*_ and *Y*^*B*^_*t.*_ *Y*^*B*^_*t* + 1_ is driven by *Y*^*B*^_*t*_ and *Y*^*D*^_*t*_ in condition 1, while *Y*^*B*^_*t* + 1_ is driven by *Y*^*A*^_*t*_*, Y*^*B*^_*t*_ and *Y*^*C*^_*t*_ in condition 2. *Y*^*D*^_*t* + 1_ is driven by *Y*^*B*^_*t*_ and *Y*^*D*^_*t*_ in condition 1, while *Y*^*D*^_*t* + 1_ is driven by *Y*^*D*^_*t*_ in condition 2. In this analysis, the conditions were predetermined by the experimental design. Our analysis of the reach-task data demonstrated that CAIM can be used for differential causal graph analysis.

## Discussion

We propose a causal discovery method called CAIM that is based on DBNs. It’s capable of revealing causal interactions among neural dynamics. Relative to static network analysis, CAIM can model complex spatiotemporal patterns of circuit activity related to a cognitive process or behavior.

We validated CAIM based on two simulated studies and a real-world spike dataset acquired during the delay period in a standard delayed reaching task. In the simulated spike train experiment, we demonstrated that CAIM accurately detected causal relationships among neuron clusters. We compared CAIM with other methods. For neuron clustering, CAIM achieved a higher Rand index than k-means and clustering by density peaks. For causal discovery, compared to BNS, BNSR, and GLMNET, CAIM achieved the optimal performance balance in AUC and running time. In the biophysics-based simulation, we generated simulated data for a set of integrate-and-fire neurons with noise. These neurons formed four clusters. CAIM accurately identified cluster structure and causal relationship between these neuron clusters. For the delayed reaching experiment, 45 neurons formed 4 clusters. The causal graphs for two different experimental conditions were different. The parent sets of nodes *A*, *B*, and *D* were different between two conditions. Collectively, these experiments demonstrated that CAIM is a powerful computation framework to detect causal relationships among neural dynamics.

The network generated by CAIM is different from that generated from synchrony analysis. Synchrony analysis centers on calculating the cross-correlation between two neural temporal courses. CAIM focuses on modeling the transition dynamics among neural temporal courses. Synchrony analysis and CAIM provide complementary information about a cognitive process.

The network model generated by CAIM is explainable; it is a graphical model and has excellent interpretability. CAIM is expandable. The computational framework in CAIM can be used for other applications such as modeling cortical traveling waves (Muller et al. 2018). Using the CAIM framework, we can detect clusters that have neurons with zero-lag synchrony; then model information propagation in a pathway and focus on the pattern that activation of cluster *A* at time point *t* leads to activation of cluster *B* at time point *t* + 1. The biophysics-based simulation provides an example of information propagation in the pathway *A* → *B* → *D*.

We have developed algorithms called dynamic network analysis to model interactions among neural signals at a macroscopic scale (Chen et al. 2012; Chen et al. 2017; Chen and Herskovits 2015). CAIM and dynamic network analysis handle different kinds of temporal data. Dynamic network analysis is designed to generate a network model from longitudinal MR data. Longitudinal MR data are short temporal sequences. For most longitudinal image data, the number of visits for each subject is small, often less than ten. Therefore, dynamic network analysis requires data from many subjects to generate a stable model, assuming that the brain network model is invariant across subjects. CAIM is designed to generate a network model from data streams which include thousands of data points. Therefore, CAIM does not assume that the brain network model is invariant across subjects.

Bayesian methods have been used to model neural activity data. Ma et al. proposed a Bayesian framework to describe how populations of neurons represent uncertainty to perform Bayesian inference (Ma et al. 2006). The probabilistic relationship between stimuli and response is formalized as P(response | stimuli). A two-layer feed-forward neural network is used for decoding. In this neural network, neurons in the output layer compute the product of input likelihood functions. Friston suggested a strong correspondence between the anatomical organization of the neocortex and hierarchical Bayesian generative models (Friston 2003). In (George and Hawkins 2009), a Bayesian model for cortical circuits is proposed. This method describes Bayesian belief propagation in a spatio-temporal hierarchical model, called hierarchical temporal memory (HTM). An HTM node abstracts space as well as time. HTM graphs use Bayesian belief propagation for inference. Deneve proposed a Bayesian neuron model in which spike trains provide a deterministic, online representation of a log probability ratio (Deneve 2005). However, the above studies about Bayesian analysis of neural activity data don’t center on causality inference.

The causal sufficiency assumption is widely used in causal discovery in order to make the causal discovery process computationally tractable. However, if there is an unmeasured time series *Z* that influences the observed time series *Y*, then the approach based on the causal sufficiency assumption can lead to incorrect causal conclusions. This is one of the limitations of CAIM. Our future research will address this limitation. We will introduce latent variables which represent unmeasured time series, then use the expectation maximization (EM) to infer properties of partially observed Markov processes (Geiger et al. 2015).

In CAIM, we assume that the causal structure is invariant across time points. If the dependencies in the underlying process change over time, the generated model is an average over different temporal dependency structures. In the future, we will extend CAIM to handle time-varying causal graphs. In this new framework, we will generate a causal graph for each time point and aggregate these causal graphs.

In the current framework, we generated a ranking of potential causal interactions. In some real-world applications, we need to determine a threshold on this ranking to obtain a binary causal graph. In future work, we will develop algorithms to overcome this challenge. One method is based on the likelihood function. For a generated binary graph, we can calculate a score to represent the likelihood that observed data is generated from the binary graph; and choose a threshold to maximize the likelihood (Chen and Herskovits 2015). This process should be inside a cross-validation procedure to avoid overfitting.

In this paper, the interactions among neural activities are represented by a 2TBN. The 2TBN represents a first-order time-invariant Markov process. We adopted the 2TBN representation to simplify the computation. In CAIM, we group neurons into clusters, effectively reducing the dimensionality of model space. An alternative approach for dimension reduction is projecting variables into a low-dimensional space and modeling dynamics among latent variables. In the future, we will develop such algorithms.

In conclusion, CAIM provides a powerful computational framework to infer causal graphs based on high-dimensional observational neural activity data. We envisage that CAIM will be of great value in understanding spatiotemporal patterns of circuit activity related to a specific behavior.

## Information Sharing Statmement

The data of the delayed reaching task is available at https://users.ece.cmu.edu/~byronyu/software/DataHigh/get_started.html. The simulated data and the software package are freely available for academic purposes on request.
